# Secretin Regulates Excitatory GABAergic Neurotransmission to GnRH Neurons via Retrograde NO Signaling Pathway in Mice

**DOI:** 10.3389/fncel.2019.00371

**Published:** 2019-08-23

**Authors:** Veronika Csillag, Csaba Vastagh, Zsolt Liposits, Imre Farkas

**Affiliations:** ^1^Laboratory of Endocrine Neurobiology, Institute of Experimental Medicine, Hungarian Academy of Sciences, Budapest, Hungary; ^2^Roska Tamás Doctoral School of Sciences and Technology, Faculty of Information Technology and Bionics, Pázmány Péter Catholic University, Budapest, Hungary; ^3^Department of Neuroscience, Faculty of Information Technology and Bionics, Pázmány Péter Catholic University, Budapest, Hungary; ^4^Laboratory of Reproductive Neurobiology, Institute of Experimental Medicine, Hungarian Academy of Sciences, Budapest, Hungary

**Keywords:** GnRH neuron, secretin, GABA, retrograde signaling, nitric oxide, reproduction, metabolism

## Abstract

In mammals, reproduction is regulated by a wide range of metabolic hormones that maintain the proper energy balance. In addition to regulating feeding and energy expenditure, these metabolic messengers also modulate the functional performance of the hypothalamic-pituitary-gonadal (HPG) axis. Secretin, a member of the secretin-glucagon-vasoactive intestinal peptide hormone family, has been shown to alter reproduction centrally, although the underlying mechanisms have not been explored yet. In order to elucidate its central action in the neuroendocrine regulation of reproduction, *in vitro* electrophysiological slice experiments were carried out on GnRH-GFP neurons in male mice. Bath application of secretin (100 nM) significantly increased the frequency of the spontaneous postsynaptic currents (sPSCs) to 118.0 ± 2.64% compared to the control, and that of the GABAergic miniature postsynaptic currents (mPSCs) to 147.6 ± 19.19%. Resting membrane potential became depolarized by 12.74 ± 4.539 mV after secretin treatment. Frequency of evoked action potentials (APs) also increased to 144.3 ± 10.8%. The secretin-triggered elevation of the frequency of mPSCs was prevented by using either a secretin receptor antagonist (3 μM) or intracellularly applied G-protein-coupled receptor blocker (GDP-β-S; 2 mM) supporting the involvement of secretin receptor in the process. Regarding the actions downstream to secretin receptor, intracellular blockade of protein kinase A (PKA) with KT-5720 (2 μM) or intracellular inhibition of the neuronal nitric oxide synthase (nNOS) by NPLA (1 μM) abolished the stimulatory effect of secretin on mPSCs. These data suggest that secretin acts on GnRH neurons via secretin receptors whose activation triggers the cAMP/PKA/nNOS signaling pathway resulting in nitric oxide release and in the presynaptic terminals this retrograde NO machinery regulates the GABAergic input to GnRH neurons.

## Introduction

Metabolic hormones report information about the actual energy state of the body to the central nervous system (CNS). Many of these hormones produced by the adipose tissue, different enteroendocrine cell populations of the gastrointestinal (GI) tract, and diverse endocrine glands, reach the hypothalamus (Berthoud, 2008), where they powerfully regulate food intake, energy expenditure, stress, water balance, and reproduction (Yamashita et al., 1989; Barash et al., 1996; Ulrich-Lai et al., 2015; Matafome et al., 2017; Hill and Elias, 2018).

In the process of reproduction, hypophysiotropic GnRH neurons located in the medial preoptic area (mPOA) of the rodent brain, are the key central regulators (Cattanach et al., 1977; Herbison, 2016). Certain metabolic hormones have already been shown to exert direct modulatory effects on GnRH neurons (Roa, 2013). Adiponectin inhibits the GnRH secretion of GT1-7 cells (Wen et al., 2008) and that of GnRH neurons of female mice (Klenke et al., 2014). Cholecystokinin (CCK) inhibits GnRH neurons directly in adult mice (Giacobini and Wray, 2007) and ghrelin, the orexigenic hormone of the stomach also decreases the firing of GnRH neurons (Farkas et al., 2013). In contrast, the anorexigenic glucagon-like peptide 1 (GLP-1) increases the activity of GnRH neurons (Farkas et al., 2016).

Secretin is an anorexigenic hormone (Thiriet, 2019), similarly to the GLP-1, and it can serve as a signal molecule reporting level of the energy homeostasis. It was the first hormone discovered in 1902 and released from the S-cells in the intestines (Bayliss and Starling, 1902). Secretin is secreted in the intestine when pylorus of the stomach opens to transfer food into the intestine. It is produced in response to the acid milieu to stimulate bicarbonate secretion from the pancreas to neutralize gastric chyme acidity. In the periphery, secretin serves, therefore, as a local signal to pancreas for neutralizing the acidity of the stomach by secretion of bicarbonates (Bayliss and Starling, 1902). In addition, those features mean that secretin can indeed be considered as a signal molecule of the high energy status of the body. Furthermore, it can cross the intact blood-brain barrier (BBB) (Banks et al., 2002; Dogrukol-Ak et al., 2004) and serve as a peripheral signal to neurons in numerous brain regions.

Secretin is also synthesized in several brain areas. The most intensive secretin immunoreactivity was detected in the Purkinje cells of the cerebellum and in some of the neurons of the deep cerebellar nuclei. Secretin immunoreactivity was also observed in a subpopulation of neurons in the primary sensory ganglia (Koves et al., 2002). Within the hypothalamus, secretin synthesis was described in the magnocellular cells of supraoptic nucleus (SON) and in the magnocellular cells of the paraventricular nucleus (PVN) (Chu et al., 2006).

The G-protein-coupled secretin receptor has a similar structure and belongs to the same receptor subfamily as the vasoactive intestinal peptide (VIP) (Ulrich et al., 1998). Specific binding of secretin to its receptor was found in various brain areas such as the cerebellum, cortex, thalamus, hippocampus, and hypothalamus (Fremeau et al., 1983). Secretin receptor mRNA showed wide distribution in the CNS. It was detected in numerous brain regions for example in the area postrema, cerebellum, central amygdala, hippocampus, thalamus, in the cortex, and in the nucleus tractus solitarii (NTS) (Nozaki et al., 2002). Dense labeling of secretin receptor was observed in the NTS and in the laterodorsal nucleus of the thalamus. Expression of secretin receptor was also found in the hypothalamus (Toth et al., 2013).

Intracerebroventricular injection of secretin increased the expression of c-Fos in several brain regions including the area postrema, medial region of the NTS, paraventricular nucleus, and various cortical areas indicating a central action of the hormone in rats. In other areas secretin attenuated c-Fos immunoreactivity (Welch et al., 2003). In the hypothalamus, intracerebroventricular administration of secretin stimulated vasopressin expression and release, indicating that it had a role in regulating the water homeostasis by modulating the hypothalamo-neurohypophysial axis (Chu et al., 2009).

Electrophysiological effect of secretin was examined first in the rat cerebellar cortex, where secretin facilitated the evoked, spontaneous, and miniature GABAergic inhibitory postsynaptic currents (IPSCs) recorded in Purkinje cells. Secretin mRNA was found in the Purkinje cells, and secretin receptor was present in both Purkinje cells and GABAergic interneurons, suggesting an autocrine regulation (Yung et al., 2001). In other electrophysiological experiments secretin depolarized neurons of the NTS via non-selective cation channels (Yang et al., 2004), while in the PVN it modulated the firing rate of the neurons *in vivo* (Chen et al., 2013).

Secretin can be regarded as another putative regulator of the reproductive axis, although limited information has been available about the exact role of secretin in the regulation of reproduction so far (Wang et al., 2018). In an early study, intracerebral (IC) injection of secretin into the preoptic region of rats resulted in 10-fold elevation of luteinizing hormone (LH) concentration in the plasma (Kimura et al., 1987). In addition, our earlier works revealed that GnRH neurons residing in the preoptic area senses the energy status of the body via various homeostatic signaling molecules such as ghrelin and GLP-1 (Farkas et al., 2013, 2016). Therefore, it is highly conceivable that secretin, as one of the signal molecules of the homeostasis, also modulates function of GnRH neurons. In addition, the anorexigenic hormone GLP-1 increased activity of GnRH neurons (Farkas et al., 2016), thus we hypothesized that secretin also stimulates GnRH neurons, promoting the reproductive process. However, the exact cellular mechanism of the effect of secretin in the modulation of HPG axis has not been revealed yet. In the present study, therefore, we carried out whole cell patch clamp recordings on GnRH-GFP neurons of male mice to elucidate the effect of secretin on PSCs, and to uncover the second messenger cascade events occurring downstream to the secretin receptor in these neurons.

## Materials and Methods

### Animals

Adult male mice were used from local colonies bred at the Medical Gene Technology Unit of the Institute of Experimental Medicine Hungarian Academy of Sciences (IEM). They were housed in light (12:12 light-dark cycle, lights on at 06:00 h)— and a temperature-controlled environment (22 ± 2°C), with free access to standard food and water. GnRH-green fluorescent protein (GnRH-GFP) transgenic mice (*n* = 57) bred on a C57Bl/6J genetic background were used for electrophysiological experiments. In this animal model, a GnRH promoter segment drives selective GFP expression in the GnRH neurons (Suter et al., 2000).

### Ethics Statement

All animal studies were carried out with permissions from the Animal Welfare Committee of the IEM Hungarian Academy of Sciences (Permission Number: A5769-01) and in accordance with legal requirements of the European Community (Directive 2010/63/EU). All animal experiments described below are designed in accord with accepted standards of animal care and all efforts were made to minimize animal suffering. We carried out sacrifice of animals by decapitation in deep anesthesia by Isoflurane inhalation.

### Brain Slice Preparation

Brain slice preparation was carried out as described earlier (Farkas et al., 2010). Briefly, after decapitation the heads were immersed in ice-cold Na-free cutting solution, continuously bubbled with carbogen, a mixture of 95% O_2_ and 5% CO_2__,_ and the brains were removed rapidly from the skull. The cutting solution contained the following (in mM): saccharose 205, KCl 2.5, NaHCO_3_ 26, MgCl_2_ 5, NaH_2_PO_4_ 1.25, CaCl_2_ 1, glucose 10. Hypothalamic blocks were dissected, and 250 μm-thick coronal slices were prepared from the medial POA with a VT-1000S vibratome (Leica Microsystems, Wetzlar, Germany) in the ice-cold Na-free oxygenated cutting solution. The slices containing POA were transferred into artificial cerebrospinal fluid (aCSF) (in mM): NaCl 130, KCl 3.5, NaHCO_3_ 26, MgSO_4_ 1.2, NaH_2_PO_4_ 1.25, CaCl_2_ 2.5, glucose 10 bubbled with carbogen and left in it for 1 h to equilibrate. Equilibration started at 33°C and it was let to cool down to room temperature.

Recordings were carried out in carbogenated aCSF at 33°C. Axopatch-200B patch-clamp amplifier, Digidata-1322A data acquisition system, and pCLAMP 10.4 software (Molecular Devices Co., Silicon Valley, CA, United States) were used for recording. Neurons were visualized with a BX51WI IR-DIC microscope (Olympus Co., Tokyo, Japan). The patch electrodes (OD = 1.5 mm, thin wall; WPI, Worcester, MA, United States) were pulled with a Flaming-Brown P-97 puller (Sutter Instrument Co., Novato, CA, United States).

GnRH-GFP neurons in the close proximity of the vascular organ of lamina terminalis (OVLT; Bregma 0.49–0.85 mm) were identified by brief illumination at 470 nm using an epifluorescent filter set, based on their green fluorescence, typical fusiform shape and characteristic topography (Suter et al., 2000).

Whole-cell patch-clamp measurements started with a control recording (5 min), then secretin was pipetted into the aCSF-filled measurement chamber containing the brain slice in a single bolus and the recording continued for a further 10 min. Pretreatment with secretin antagonist (1 μM) started 10 min before adding the secretin and the antagonist was continuously present in the aCSF during the electrophysiological recording. Intracellularly applied drugs, such as the membrane impermeable G-protein inhibitor GDP-β-S (2 mM, Sigma; St. Louis, MO, United States), NO synthase inhibitor NPLA (1 μM; Tocris; Bristol, United Kingdom), and PKA blocker KT-5720 (2 μM; Tocris) were added to the intracellular pipette solution and after achieving whole-cell patch clamp configuration, we waited 15 min to reach equilibrium in the intracellular milieu before starting recording. Each neuron served as its own control when drug effects were evaluated.

### Reagents and Chemicals

#### Extracellularly Used Drugs

Secretin (30 nM–1 μM; rat, Tocris); Secretin antagonist [3 μM; Secretin 5–27; TFTSELSRLQDSARLQRLLQGLV (Williams et al., 2012)], GABA_A_-R blocker picrotoxin [100 μM, Sigma; (Seidl et al., 2014; Keshavarzi et al., 2015)].

#### Intracellularly Used Drugs

Neuronal nitric oxide synthase (nNOS) inhibitor N-propyl-L-arginine hydrochloride [NPLA; 1 μM; Tocris (Chow et al., 2012; Filpa et al., 2015; Gong et al., 2015)]; G-protein inhibitor, Guanosine 5′-[β;-thio] diphosphate [GDP-β-S; 2 mM; Sigma, (Meis et al., 2002; Ponzio and Hatton, 2005; McDermott and Schrader, 2011)]; protein kinase-A (PKA) inhibitor (9S,10S,12R)-2,3,9,10,11,12-Hexahydro-10-hydroxy-9-methyl-1-oxo-9,12-epoxy-1H-diindolo[1,2,3-fg:3′,2′,1′-kl]pyrrolo[3,4-i][1,6]benzodiazocine-10-carboxylic acid hexyl ester [KT-5720; 2 μM; Tocris (Glovaci et al., 2014; Kaneko et al., 2016)].

### Whole Cell Patch Clamp Experiments

The spontaneous postsynaptic currents (sPSCs) and miniature postsynaptic currents (mPSCs) in GnRH neurons were measured as described earlier (Farkas et al., 2010). Briefly, the neurons were voltage-clamped at –70 mV holding potential. Intracellular pipette solution contained (in mM): HEPES 10, KCl 140, EGTA 5, CaCl_2_ 0.1, Mg-ATP 4, Na-GTP 0.4 (pH = 7.3 with NaOH). The resistance of the patch electrodes was 2–3 MΩ. Spike-mediated transmitter release was blocked in all mPSC experiments by adding the voltage-sensitive Na-channel inhibitor tetrodotoxin (TTX, 660 nM, Tocris) to the aCSF 10 min before mPSCs or V_rest_ were recorded. The mPSCs recorded under the conditions used in our experiments were related to GABA_A_-R activation (Sullivan et al., 2003; Farkas et al., 2010). This GABAergic input was also validated in our measurements by picrotoxin (100 μM, Tocris). GABAergic input via GABA_A_-R is excitatory to GnRH cells (Moenter and DeFazio, 2005; Yin et al., 2008; Herbison and Moenter, 2011). Time distribution graphs of frequencies were generated by using 1-minute time bins to show time courses of effect of secretin.

Resting membrane potential (V_rest_) was recorded in current-clamp mode with 0 pA holding current. To show action of secretin on the firing, R_in_, and C_m_ in GnRH neurons of male mice, current clamp measurements were recorded. Three 900-ms-long current steps were applied (−25, 0, and +25 pA). Firing was analyzed during the depolarizing step. The R_in_ was determined from the voltage response to the application of hyperpolarizing current. The time constant was the time required to reach 63% of the maximum voltage response to hyperpolarizing current (Spergel et al., 1999). The C_m_ was then calculated by dividing the time constant by the R_in_. After control recording, secretin was pipetted into the measurement chamber and 1, 3, 5, and 10 min later the three current steps were repeated.

### Statistical Analysis

Recordings were stored and analyzed off-line. Event detection was performed using the Clampfit module of the PClamp 10.4 software (Molecular Devices Co., Silicon Valley, CA, United States).

Spontaneous postsynaptic currents and mPSC frequencies were calculated as number of PSCs divided by the length of the corresponding time period (5 or 10 min). Mean values of the control and treated part of the recording were calculated from these frequency values. All the experiments were self-controlled in each neuron: percentage changes in the parameters of the PSCs were calculated by dividing the value of the parameter in the treated period with that of the control period.

Evoked AP frequency was calculated by dividing the number of events with the length of the respective time period. Percentage changes resulted from secretin application were calculated by dividing the value to be analyzed before and after secretin administration.

Group data were expressed as mean ± standard error of mean (SEM). Two-tailed Student’s *t* test were applied for comparison of groups and the differences were considered as significant at *p* < 0.05. Cumulative probabilities of interevent-intervals of neurons were analyzed by using Kolmogorov–Smirnov test (*p* < 0.05) to show statistical differences between the interevent-intervals of the control and secretin treated periods. The analysis of frequency changes in case of the evoked action potentials (APs) was carried out by One-way ANOVA with repeated measurements followed by Dunnett’s test.

## Results

### Secretin Increased the Frequency of Spontaneous Postsynaptic Currents and Depolarized the Membrane Potential in GnRH Neurons of Male Mice

**FIGURE 1:**
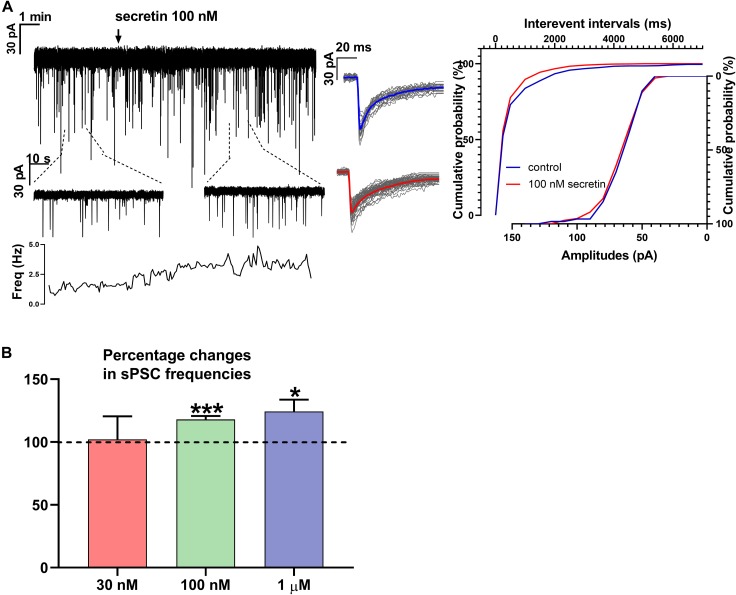
Secretin increases the frequency of sPSC in GnRH neurons. **(A)** Secretin increased the frequency of the sPSCs with no change in the average amplitude and shape. Average sPSCs next to the recording represent no change in the shape of events after secretin treatment. The inserts below the 15 min recordings are 1-1 min zoomed periods from the recordings before and after secretin administration. The frequency distribution graph under the inserts also reveals that secretin application elevated the sPSC frequency. Cumulative probabilities of the interevent intervals and amplitudes are also presented. **(B)** The bar graph shows that effect of secretin increasing the sPSC frequency is dose dependent (Student’s *t*-test ^∗^*p* < 0.05; ^∗∗∗^*p* < 0.001).

Administration of 30 nM secretin revealed no significant change neither in frequency (Figure 1B) nor in amplitude parameters of sPSCs. Rise and decay τ of sPSCs also presented no significant change (Tables 1, 2, 5).

**TABLE 1:** Changes in the frequency of PSCs after secretin treatment.

		**Frequency of control period in Hz**	**After secretin in Hz**	**Average percentage changes after secretin**	***n*/N**
**sPSC**	**30 nM secretin**	1.348 ± 0.442	1.232 ± 0.235	102.10 ± 4.33	9/4
	**100 nM secretin**	3.244 ± 0.8155	3.768 ± 0.958	118 ± 2.64^∗∗∗^	8/3
	**1 μM secretin**	1.914 ± 0.509	2.413 ± 0.6484	124.3 ± 9.404^∗^	7/3
**mPSC**	**100 nM secretin**	1.367 ± 0.315	1.833 ± 0.7176	147.6 ± 19.19^∗^	16/7
	**Secretin receptor antagonist + 100 nM secretin**	0.7229 ± 0.2358	0.566 ± 0.115	92.88 ± 8.949	8/4
	**GDP-β-S + 100 nM secretin**	0.632 ± 0.124	0.6090 ± 0.1199	102.1 ± 0.957	10/4
	**NPLA + 100 nM secretin**	1.045 ± 0.2297	1.018 ± 0.2380	90.38 ± 4.60	10/5
	**KT5720 + 100 nM secretin**	2.016 ± 0.7367	1.755 ± 0.5721	97 ± 5.987	13/6

*The first column shows the frequency of the control period in Hz. Second and third columns show frequency change after secretin and inhibitor pretreatments. The fourth column shows the number of neurons (*n*) and animals (N) used for the experiments (Student’s *t*-test; ^∗^*p* < 0.05; ^∗∗∗^*p* < 0.001).*

**TABLE 2:** Changes in the amplitude of PSCs upon secretin treatment.

		**Amplitude of control period in pA**	**Average percentage changes after secretin**	***n*/N**
**sPSC**	**30 nM secretin**	31.98 ± 3.628	96.78 ± 3.166	9/4
	**100 nM secretin**	54.04 ± 7.366	97.38 ± 1.209	8/3
	**1 μM secretin**	37.91 ± 2.727	99.71 ± 3.160	7/3
**mPSC**	**100 nM secretin**	41.77 ± 3.061	101.3 ± 2.406	16/7
	**Secretin receptor antagonist + 100 nM secretin**	47.75 ± 3.034	100.3 ± 2.295	8/4
	**GDP-β-S + 100 nM secretin**	76.56 ± 9.424	101.0 ± 1.483	10/4
	**NPLA + 100 nM secretin**	38.95 ± 3.347	104.1 ± 4.037	10/5
	**KT5720 + 100 nM secretin**	54.04 ± 9.231	99.0 ± 1.665	13/6

*The first column shows the amplitude of the PSCs (pA) in control period. Second column shows changes in amplitudes in%, the third column shows the number of neurons (*n*) and animals (N) used for the experiments.*

Frequency of sPSCs after 100 nM secretin administration resulted in a significant increase up to 118.0 ± 2.64% of the control values (3.244 ± 0.8151 Hz, *n* = 8, Student’s *t*-test, *p* = 0.0005) (Figures 1A,B and Table 1). The increase in frequency of the sPSCs started approximately 2 min after the administration of secretin, as shown by the distribution graph under the recording (Figure 1A). Cumulative probability plots also demonstrated significant difference between the control and the treated interevent-intervals (Kolmogorov–Smirnov test, *p* = 0.0047). In contrast, values of amplitude, rise, and decay τ of the sPSCs presented no significant change (Figure 1A and Tables 2, 5).

Administration of 1 μM secretin also significantly increased the frequency of sPSCs to 124.3 ± 9.404% (control value: 1.914 ± 0.519 Hz, Student’s *t*-test, *n* = 7, *p* = 0.049) (Figure 1B and Table 1). The bar graph shows the percentage changes in the frequency of sPSCs resulted from secretin application, demonstrating the dose dependency of the effect of secretin (Figure 1B). Values of amplitude, rise, and decay τ of the sPSCs after 1 μM secretin administration presented no significant change (Tables 2, 5).

### Secretin Increased the Frequency of Evoked Action Potentials in GnRH Neurons of Male Mice

**FIGURE 2:**
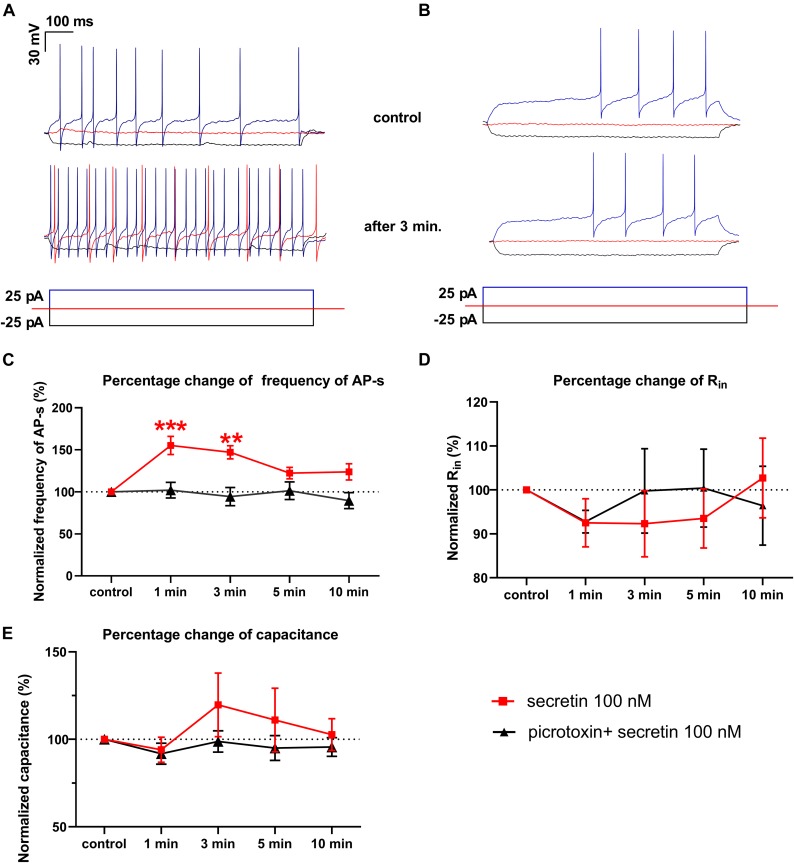
Secretin (100 nM) increases the frequency of the evoked APs. **(A)** Representative recording shows that frequency of APs evoked by depolarizing current steps elevated 3 min after secretin administration. Also, the rheobase of APs decreased after secretin treatment. There was no change in the average amplitudes of APs. **(B)** Representative recording of the effect of secretin in the presence of picrotoxin **(C)** Secretin results in a significant rise in the frequency of APs after 1 and 3 min of its administration (marked by red ■) in the presence of picrotoxin there was no significant change (marked by ▲). **(D)** Changes in the R_in_ represented no significant alteration. **(E)** Membrane capacitance also showed no significant change. (^∗∗^*p* < 0.01; ^∗∗∗^*p* < 0.001).

The number of evoked APs increased significantly after secretin administration (100 nM) when measured in current clamp mode in 1 and 3 min time points. The frequency increased after 1 min to 144.3 ± 10.8% (*p* = 0.0005) and after 3 min up to 138.2 ± 11.24% compared to the control value [11.56 ± 1.819 Hz (*p* = 0.0023)]. Firing rate showed no significant changes at other time points (Figures 2A,C and Table 3) (*n* = 7; One-way ANOVA with repeated measurements).

**TABLE 3:** Effect of secretin on the evoked action potentials (APs), the passive membrane properties and V_rest_.

	**Control**	**Average percentage or delta changes after secretin**				
			
		**1 min**	**3 min**	**5 min**	**10 min**	***n*/N**
**Frequency of APs**	11.56 ± 1.819*Hz*	144.3 ± 10.8%*⁣**	138.2 ± 11.24%**	114.7 ± 9,45%	123.7 ± 9.76%	7/3
**Capacitance**	22.66 ± 3.93*pF*	97.57 ± 6.09%	100.6 ± 7.387%	106.4 ± 6.244%	109.6 ± 7.422%	7/3
**R_in_**	972 ± 96.13*M*Ω	92.5 ± 5.476%	92.33 ± 7.54%	93.5 ± 6.756%	102.7 ± 9.087%	7/3
**V_rest_**	70.61 ± 4.498*mV*	11.92 ± 4.487*mV*^∗∗^	13.82 ± 4.986*mV*^∗∗^	10.37 ± 8.052*mV*	8.540 ± 6.795*mV*	7/3

*The first column shows the control values of the frequency, capacitance, input resistance (R_*in*_) and the V_*rest*_. The next four columns demonstrate the changes in the measured parameters after 1, 3, 5, and 10 min. The last column contains the number of neurons (*n*) and animals (N) used for the experiments (One-way ANOVA with repeated measurements followed by Dunnett’s test ^∗∗^*p* < 0.01; ^∗∗∗^*p* < 0.001).*

The rheobase, which shows the strength of the current required to activate a single action potential, decreased in 6 neurons out of 7, after application of secretin. Firing in 3 neurons of 7 started at 0 pA current injection, suggesting that secretin could increase the spontaneous activity of these neurons. Other passive membrane parameters, such as input resistance (R_in_) and membrane capacitance (C_m_) also showed no significant change (Figures 2D,E and Table 3). The current step measurements showed, that resting membrane potential (V_rest_) depolarized significantly at 1 and 3 min (11.92 ± 4.487 mV, *p* < 0.0451; 13.82 ± 4.986 mV, *p* < 0.0392, Table 3) suggesting that elevation in the firing rate resulted from this change in the V_rest_.

Earlier studies showed that in GnRH neurons of adult male mice the mPSCs are exclusively excitatory through GABA_A_ receptor (Sullivan et al., 2003; Sullivan and Moenter, 2004; Moenter and DeFazio, 2005; Farkas et al., 2010; Herbison and Moenter, 2011). We eliminated all the mPSCs by application of selective GABA_A_ receptor blocker picrotoxin and after secretin administration no new PSCs could be observed (not shown) suggesting that the recorded mPSCs in these experiments were GABA_A_ receptor-mediated currents.

We hypothesized that GABA_A_ receptor plays an exclusive role in the effect of secretin on the firing of GnRH neurons. The GABA_A_-R blocker picrotoxin totally eliminated the effect of secretin on the evoked APs of GnRH neurons, there was no residual change (Figures 2B,C). This fact indicates that effect of secretin on the firing rate correlates with the action of secretin on the GABAergic PSC frequency, and the elevation in the firing rate (i.e., the increased excitability) results from the elevated frequency of the GABAergic PSCs. Other passive membrane parameters, such as R_in_ and C_m_ also showed no significant change (Figures 2D,E and Table 4). The input resistance is the sum of the membrane resistance and the electrode resistance (Barbour, 2014). Supposing that electrode resistance does not change during the measurement, the input resistance is a true measure of the membrane resistance. Therefore, if the input resistance shows no change it indicates that membrane resistance presents no change, too. The current step measurements also showed, that in the presence of picrotoxin V_rest_ presented no significant change at any time point (Table 4) indicating that GABAergic neurotransmission plays role in the membrane depolarization which eventually results in the elevation in the firing rate.

**TABLE 4:** Effect of secretin on the evoked APs, the passive membrane properties and V_rest_ in the presence of picrotoxin.

	**Control**	**Average percentage or delta changes after secretin**				
			
		**1 min**	**3 min**	**5 min**	**10 min**	***n*/N**
**Frequency of APs**	15.18 ± 6.878*Hz*	102 ± 9.295%	94.40 ± 10.79%	101.4 ± 10.50%	89.60 ± 9.51%	6/3
**Capacitance**	18.87 ± 0.8304*pF*	91.80 ± 6.012%	98.80 ± 6.094%	95.0 ± 7.162%	95.60 ± 5.354%	6/3
**R_in_**	1048 ± 116.6*M*Ω	92.80 ± 2.296%	99.80 ± 9.604%	100.4 ± 8.875%	96.40 ± 8.976%	6/3
**V_rest_**	65.14 ± 5.042*mV*	0.5075 ± 0.4413*mV*	0.3663 ± 0.3472*mV*	0.8924 ± 0.2314*mV*	1.5970 ± 0.7586*mV*	6/3

*The first column shows the control values of the frequency, capacitance, input resistance (R_*in*_) and V_*rest*_. The next four columns demonstrate the changes in the measured parameters after 1, 3, 5, and 10 min. The last column contains the number of neurons (*n*) and animals (N) used for the experiments (One-way ANOVA with repeated measurements followed by Dunnett’s test).*

### Secretin Acted Directly on GnRH Neurons via Secretin Receptor

**FIGURE 3:**
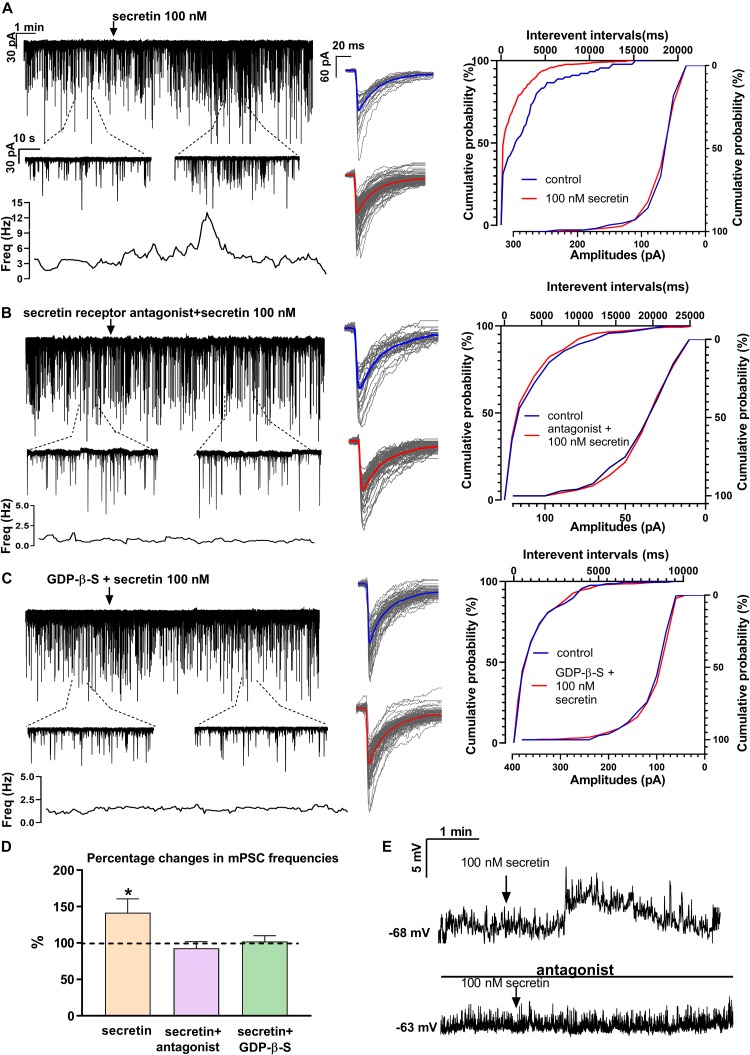
Secretin elevates the frequency of mPSCs of GnRH neurons directly via secretin receptor. **(A)** Secretin (100 nM) increased the frequency of mPSCs in GnRH neurons, as shown in a representative recording, the 1 min zoomed periods, the frequency distribution and the cumulative probability of IEIs graphs. There was no change in the average amplitude or in the shape of the events representing the individual PSCs beside the recording. **(B)** Pretreatment of the brain slice with secretin receptor antagonist (Secretin 5–27) eliminated the effect of secretin on GnRH neurons. **(C)** Intracellular application of G-protein blocker, GDP-β-S also abolished the effect of secretin. **(D)** Bar graph shows that the effect of secretin was mediated via the G-protein coupled secretin receptor. **(E)** Depolarization in the resting potential is demonstrated in a 4 min period. Arrow shows application of secretin. The bottom recording, in the presence of secretin receptor antagonist shows no significant change after the administration of secretin (100 nM) (^∗^*p* < 0.05). The inserts below the 15 min recordings are 1-1 min zoomed periods from the recordings before and after secretin administration. The frequency distribution is also presented under each recording. Average mPSCs next to the recording represent no change in the shape of events after secretin treatment. Cumulative probabilities of the interevent intervals and amplitudes are graphed next to the individual events. Arrow shows the administration of secretin (^∗^*p* < 0.05).

In order to demonstrate the direct action of secretin on GnRH neurons, mPSCs were recorded in the presence of TTX. The administration of secretin (100 nM) resulted in a significant increase in the mean mPSC frequency reaching 147.6 ± 19.19% of control values (1.367 ± 0.315 Hz, *n* = 16; Student’s *t*-test; *p* = 0.0274) (Figure 3A and Table 1). Elevation of the mPSC frequency started 1–3 min after administration of secretin. Cumulative probability plots also demonstrated significant differences between the control and the treated interevent-intervals (Kolmogorov–Smirnov test, *p* = 0.0337). Values of amplitude, rise τ, and decay τ of the mPSCs presented no significant change (Figure 3A and Tables 2, 5).

**TABLE 5:** Changes in the rise τ and decay τ after 100 nM secretin.

		**Rise tau (ms)**	**Average percentage changes after secretin**	**Decay tau (ms)**	**Average percentage changes after secretin**	***n*/N**
**sPSC**	**30 nM secretin**	3.716 ± 0.440	103.9 ± 4.808	22.57 ± 1.431	102.8 ± 4.160	9/4
	**100 nM secretin**	4.460 ± 0.6166	101.6 ± 7.926	23.05 ± 1.711	97.88 ± 3.182	8/3
	**1 μM secretin**	4.244 ± 0.3608	98.38 ± 5.867	26.17 ± 1.833	101.9 ± 1.903	7/3
**mPSC**	**100 nM secretin**	4.866 ± 0.6075	101.7 ± 12,96	19.07 ± 2.140	104.6 ± 12.18	16/7
	**Secretin receptor antagonist + 100 nM secretin**	4.081 ± 0.8153	102.8 ± 7.947	32.78 ± 10.22	97.30 ± 10.82	8/4
	**GDP-β-S + 100 nM secretin**	3.940 ± 0.4759	100.03 ± 10.29	22.96 ± 4.250	91.20 ± 7.297	10/4
	**NPLA + 100 nM secretin**	3.281 ± 0.453	106.3 ± 8.593	21.51 ± 4.044	102.4 ± 6.608	10/5
	**KT5720 + 100 nM secretin**	4.475 ± 0.4489	101.8 ± 10.61	20.26 ± 1.258	112.5 ± 10.02	13/6

*The first column shows the rise tau in the control period. The second column shows changes in the rise time in%. Third column shows the decay tau in the control period, the fourth column shows changes in the decay time in%. The fifth column shows the number of neurons (*n*) and animals (N) used for the experiments.*

Pretreatment of the slices with secretin receptor antagonist (secretin 5–27; 3 μM) 15 min before the application of secretin (100 nM), eliminated the stimulatory action of secretin on the mean frequency of mPSCs (92.88 ± 8.949%) (Figure 3B and Table 1). Cumulative probability plots also showed no significant differences between the control and the treated interevent-intervals (Kolmogorov–Smirnov test, *p* = 0.999). Values of mPSC amplitude, rise and decay τ presented no significant change (Figure 3B and Tables 2, 5).

In order to prove the direct action of secretin in GnRH neurons, its effect on the mPSCs was further examined in the intracellular presence of the G-protein blocker GDP-β-S (2 mM). The blockade of G-proteins in GnRH neurons eliminated the observed effect of secretin on mPSCs (102.1 ± 0.957%) (Figure 3C and Table 1). Cumulative probability plots showed no significant differences between the control and the treated interevent-intervals (Kolmogorov–Smirnov test, *p* = 0.819). Values of amplitude and shape of the PSCs also presented no significant changes (Figure 3C and Tables 2, 5).

Bar graph summarizes the effect of secretin on the mean frequency of the mPSCs and full inhibition of the secretin-triggered action by antagonizing secretin receptor and the intracellular blockade of G-proteins in GnRH neurons (Figure 3D and Table 1).

Current clamp measurements revealed that secretin (100 nM) triggered membrane depolarization in GnRH neurons in the presence of TTX. The mean of the changes was 12.74 ± 4.539 mV (Student’s *t*-test, *n* = 6, *p* = 0.0186) (Figure 3E). Depolarization usually occurred 1 min after secretin application and as the figure shows it returned to the baseline after a short time. In the presence of secretin receptor antagonist, the observed stimulating effect did not occur, there was no significant change in the membrane potential (*n* = 5) (Figure 3E).

### Involvement of PKA and Retrograde NO Signaling Mechanisms in the Effect of Secretin

**FIGURE 4:**
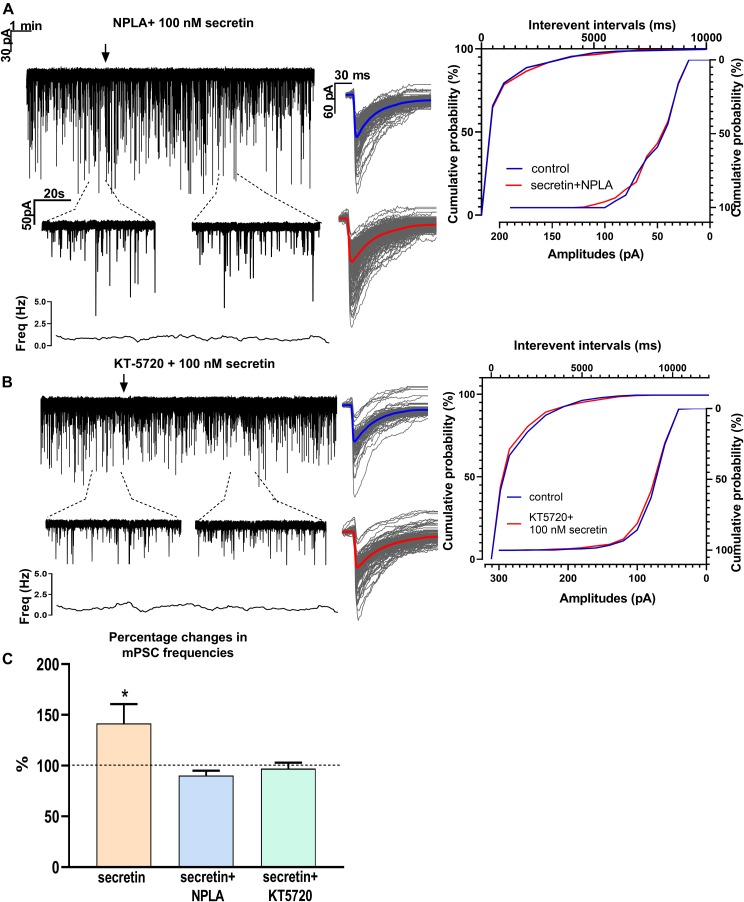
Effects of various blockers on the secretin-evoked increase in the frequency of mPSCs in GnRH neurons. **(A)** Intracellular application of the nNOS blocker NPLA extinguished the effect of secretin. **(B)** The PKA inhibitor KT5720 applied intracellularly, also eliminated the effect of secretin. **(C)** Bar graph shows that secretin utilizes PKA- and retrograde NO-coupled signaling mechanisms. The inserts below the 15 min recordings are 1-1 min zoomed periods from the recordings before and after secretin administration. The frequency distribution is also presented under each recording Average mPSCs beside each recording showed change in the shape or amplitudes of events after secretin treatment. Cumulative probabilities of the interevent intervals are graphed next to the individual events. Arrow shows the administration of secretin (^∗^*p* < 0.05).

Changes in the frequency of mPSCs but not in the amplitude suggested changes in the presynaptic site after application of secretin. Previous studies have demonstrated that activation of the retrograde NO signaling pathway in GnRH neurons results in an increased mPSC frequency (Farkas et al., 2016). To test the hypothesis that retrograde NO signaling mediates the effect of secretin on the GABAergic synaptic input of GnRH neurons NPLA (1 μM), an nNOS blocker, was applied intracellularly into the recorded GnRH neurons 15 min before adding secretin (100 nM). Intracellular application of NPLA alone did not alter basal frequency or amplitude of mPSCs in GnRH neurons (Farkas et al., 2016; Figure 4A and Table 1). NPLA treatment fully eliminated the action of secretin (90.38 ± 4.60%, Student’s *t*-test, *p* = 0.0746). Cumulative probability plots showed no significant differences between the control and the treated interevent-intervals (Kolmogorov–Smirnov test, *p* = 0.998). Values of amplitude and rise and decay τ of the PSCs also presented no significant changes (Figure 4A and Tables 2, 5).

Nitric oxide activation can be induced via different intracellular signaling pathways. Earlier studies showed that one of the main pathways activated by secretin receptor is the cAMP/PKA pathway (Siu et al., 2006). Therefore, the selective PKA blocker KT5720 was applied intracellularly into GnRH neurons. The presence of KT5720 in the intracellular solution abolished the frequency increasing effect of secretin on mPSCs of GnRH neurons (97 ± 5.987%) (Figure 4B and Table 1). Cumulative probability plots represented no significant differences between the control and the treated interevent intervals (Kolmogorov–Smirnov test, *p* = 0.491). Values of amplitude, the rise and the decay τ of mPSCs also presented no significant changes (Figure 4B and Tables 2, 5). Bar graph depicts the full inhibition of the secretin-triggered action on mPSCs by intracellularly applied NPLA and KT5720 (Figure 4C and Table 1).

**FIGURE 5:**
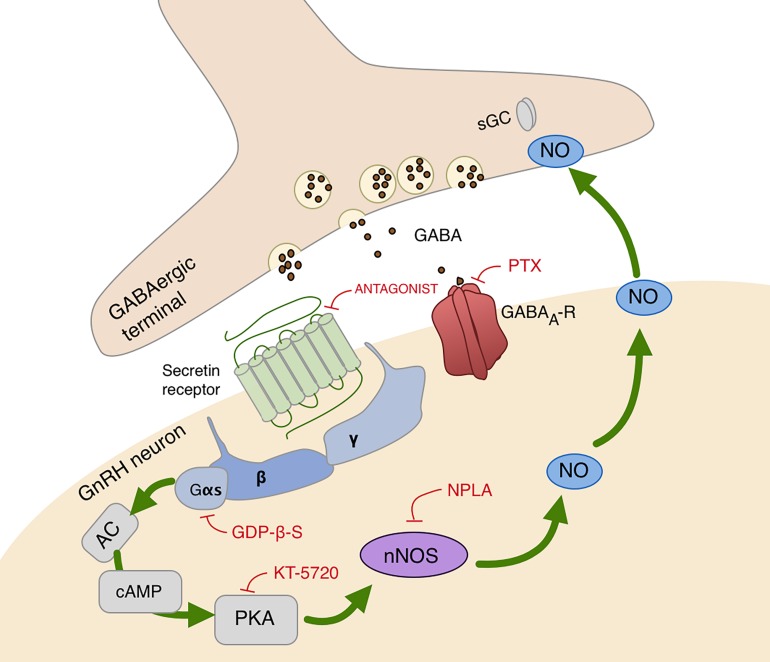
Schematic illustration of secretin receptor signaling in GnRH neurons. Secretin activates cAMP/PKA/nNOS pathway and generates NO that binds to its presynaptic receptor, sGC, located in the GABAergic terminals. This signaling process increases the release of GABA, therefore, facilitates the synaptic inputs to GnRH neurons via GABA_A_-receptor. AC, adenylate cyclase; cAMP, cyclic adenosine monophosphate; Gαs, Gβ, Gγ, G-protein subunits; GABA_A_-R, GABA_A_-receptor; PTX, picrotoxin, selective GABA_A_-receptor blocker; PKA, protein kinase A; KT5720, protein kinase A inhibitor; nNOS, neuronal nitric oxide synthase; NPLA, nNOS inhibitor; GDP-β-S,G-protein inhibitor; sGC, soluble guanylyl cyclase, NO receptor. Red lines depict inhibitory actions, green arrows refer to the signal transduction pathway resulting in excitatory action of NO.

In summary, these results demonstrate that secretin acts directly on GnRH neurons via secretin receptors and activates the cAMP/PKA/nNOS pathway which enables the generation of NO in the recorded GnRH neurons in male mice. The retrograde messenger can reach the GABAergic synaptic boutons and increases the release of GABA enhancing frequency of GABAergic mPSCs of GnRH neurons as seen on the schematic illustration (Figure 5).

## Discussion

Secretin has been shown to modulate the HPG axis, however, the sites of action have not been explored so far (Kimura et al., 1987). The present study provides electrophysiological evidence for a direct action of secretin on GnRH neurons. Accordingly: (1) Secretin activated the secretin receptors directly in GnRH neurons and increased the frequency of their APs and GABAergic mPSCs. (2) Downstream signaling of secretin receptor involved the activation of PKA and nNOS that in turn, led to activation of the retrograde NO signaling pathway. The release of NO enhances the release of GABA from the presynaptic sites which has an excitatory effect on GnRH neurons via GABA_A_-R.

### Secretin Is Excitatory on GnRH Neurons via Secretin Receptor

The present findings demonstrate that secretin exerts excitatory effects on GnRH neurons. Frequency of PSCs and APs increased, the rheobase of evoked APs decreased, and the membrane potential depolarized upon secretin administration. These data are parallel with other investigations showing the stimulatory effect of secretin in different brain areas and different cell types. In the PVN secretin elevated the firing rate *in vivo*, and in the nucleus of the solitary tract (NTS) it depolarized neurons via non-selective cationic channels (Yang et al., 2004; Chen et al., 2013). Both central and peripheral administration of secretin induced c-Fos expression in the PVN and the arcuate nucleus, suggesting the activation of these hypothalamic nuclei (Cheng et al., 2011).

Both the GABAergic mPSC frequency and the firing rate were increased in GnRH neurons after secretin administration. Since GABA has a special excitatory effect on GnRH neurons via GABA_A_-R (Sullivan et al., 2003; Farkas et al., 2010), the elevation detected in mPSC frequency and the increased firing rate correlate well.

Although the recorded mPSCs of GnRH neurons in male mice are GABAergic under basal conditions, we could not exclude the theoretical possibility of the additional effects of glutamate on these parameters (Sullivan and Moenter, 2003; Moenter and DeFazio, 2005; Yin et al., 2008; Farkas et al., 2010; Herbison and Moenter, 2011). The selective inhibition of GABA_A_ receptors with picrotoxin, however, totally abolished the effect of secretin on PSCs, indicating the exclusive role of GABAergic inputs in the effects of secretin.

We proved that the effect of secretin is specific to secretin receptor using secretin receptor antagonist. In addition, the intracellular blockade of secretin receptor by the membrane impermeable G-protein blocker, GDP-β-S, also abolished the secretin-evoked changes in the mPSC frequency, proving that secretin action occurs postsynaptically on GnRH neurons.

A previous study has reported that secretin augmented plasma LH concentration following its stereotaxic delivery into mPOA suggesting that the effect of secretin on LH cells and LH production was indirect, and presumably the activation occurred at the level of GnRH neurons (Kimura et al., 1987). Our findings confirm that secretin is capable of centrally regulating the HPG axis via a direct activation of GnRH neurons.

Changes in the frequency of GABAergic mPSCs but not in their amplitude suggest that alterations take place at the presynaptic site. This might indicate that secretin has a direct effect at the presynaptic site via secretin receptors as it was discovered in an earlier work from the Purkinje cells of cerebellum (Yung et al., 2001). In our study, the intracellular blockade of the G-proteins and the NO pathway in the postsynaptic GnRH neurons eliminated the effect of secretin excluding this opportunity.

The hyperpolarizing current step measurements showed that input resistance (and therefore membrane resistance) has not changed upon secretin application and the subsequent GABA release. This seems to be in contrast with the data revealing a secretin-dependent depolarization of V_rest_ when the current step is zero. One of the putative explanations for this observation is that response to GABA can be voltage-dependent, provided presence of GABA_B_-R in the neuron (Shefner and Osmanovic, 1991). It is well known that GnRH neurons bear GABA_B_-R (Herbison and Moenter, 2011; Liu and Herbison, 2011) suggesting a putative voltage-dependency of GABA response in GnRH neurons. This opportunity, however, requires further elaboration.

### Secretin Activates the Retrograde NO Signaling Pathway via cAMP/PKA Upregulation

Our results indicate that secretin acts directly on GnRH neurons via secretin receptors whose activation triggers a downstream cascade event leading to activation of retrograde NO signaling in GnRH neurons. The mechanism of secretin’s effect is often linked to NO production at the periphery (Konturek et al., 1997; Jyotheeswaran et al., 2000; Grossini et al., 2013). Within the brain, increase in the frequency of the GABAergic mPSCs could be evoked by activation of the NO machinery in hypothalamic neurons (Di et al., 2009). Previously, the presence and the activity of nNOS have been demonstrated by our group in GnRH neurons of mice. In these studies, the activation of nNOS increased the frequency of GABAergic mPSCs in GnRH neurons (Farkas et al., 2016, 2018). Stimulatory effect of retrograde NO signaling on GABAergic currents was described earlier in the PVN, where NO excited the GABAergic mPSCs in the neurons of PVN (Bains and Ferguson, 1997). NO also activated the GABAergic inputs of oxytocin- and vasopressin-containing neurons in the SON (Stern and Ludwig, 2001). The stimulatory effect of retrograde NO signaling was also proved in case of glutamatergic neural circuits, where NO released from the postsynaptic neurons could increase the probability of glutamate release from presynaptic glutamatergic axon terminals in different brain areas (O’Dell et al., 1991; Arancio et al., 1996; Micheva et al., 2003; Neitz et al., 2011).

All members of the B1 family of G-protein coupled receptors such as, VIP, secretin, and pituitary adenylate cyclase-activating peptide (PACAP) stimulate the adenylate cyclase via stimulatory G-protein (Gs) (Roth et al., 1984; Fremeau et al., 1986; Harmar, 2001) resulting in PKA activation (Siu et al., 2006) via Gs protein. The presence of PKA in GnRH secreting neurons was found earlier (Hoddah et al., 2009), and we also revealed that the application of PKA blocker totally inhibited the effect of secretin, providing further evidence that the underlying mechanism of secretin requires PKA activation.

The present findings demonstrate that secretin acted postsynaptically and resulted in a change of mPSC frequency, suggesting that secretin receptor activates the cAMP/PKA/nNOS pathway and generates NO that binds to its presynaptic receptor, soluble guanylyl cyclase (sGC), located in the GABAergic axon terminals. The expression of sGC has been recently detected in GABAergic and glutamatergic presynaptic boutons of GnRH neurons (Farkas et al., 2016, 2018). This retrograde signaling process increases the release of GABA, therefore, facilitates the synaptic inputs to GnRH neurons via GABA_A_-receptor. PACAP and VIP can also activate nNOS and the production of NO via the cAMP/PKA/nNOS pathway in PC12 cells (Onoue et al., 2002).

These current results support further the concept that GnRH neurons can sense the metabolic state of the organism and strengthen the view that secretin may exert a central regulatory role on the HPG axis via acting upon hypophysiotropic GnRH neurons.

## Data Availability

All datasets generated for this study are included in the manuscript/supplementary files.

## Author Contributions

VC wrote the manuscript and carried out electrophysiological measurements. CV and ZL designed the experiments and wrote the manuscript. IF designed the experiments, wrote the manuscript and carried out electrophysiological measurements.

## Conflict of Interest Statement

The authors declare that the research was conducted in the absence of any commercial or financial relationships that could be construed as a potential conflict of interest.
